# Toward a comparative understanding of beat perception and synchronization

**DOI:** 10.1186/s12868-024-00852-5

**Published:** 2024-11-06

**Authors:** Marc F Schmidt, Michael Kaplan

**Affiliations:** 1 Department of Biology, University of Pennsylvania, 19104 Philadelphia, PA USA; 2 Neuroscience Program, University of Pennsylvania, 19104 Philadelphia, PA USA

## Abstract

BPS provides unique opportunities for comparative studies to understand the mechanism(s) of beat perception and its translation into synchronous motor output. We argue that an evolutionary perspective of this behavior will require a clearer dissociation between beat perception and synchronization and a more parameterized approach to studying this behavior.

**Supplementary Information:**

The online version contains supplementary material available at 10.1186/s12868-024-00852-5.

## Commentary

In this interesting review, Patel proposes the types of neural circuit modifications that might have allowed humans and parrots the rare ability to synchronize their movements to a musical beat– that is, to dance. Key to his argument is that the ability to translate beat perception into synchronized motor output (such as in the form of tapping or dancing) should require strong, temporally precise bi-directional connectivity between auditory regions that can entrain to periodic rhythms and premotor areas responsible for this motor output. The observation that parrots, which, like humans, are vocal learners, are also able to move rhythmically to beat-based music, suggests the hypothesis that vocal learning might be necessary for beat perception and synchronization (BPS). There are of course many species capable of vocal learning and many, if not most, of these do not show BPS even though arguably even more have not been tested (including many species of birds, bats and cetaceans). Nevertheless, it seems that the ability for vocal learning may indeed be a necessary if not sufficient condition for beat perception to manifest as motor synchronization. This remarkable behavior opens wonderful opportunities for comparative studies aimed at dissecting the neural mechanisms that allow beat perception, ask how rhythmic responses are translated into synchronous motor output and ultimately gain insight into why this behavior evolved.

A general assumption in the ability of pet parrots to perform BPS is that it arises spontaneously without dependence on formal training or physical rewards. While suggestive, evidence to support these claims is not strong enough to rule out that parrots do not require some form of learning or reinforcement. We caution that over-reliance of this assumption might cloud our view of the possible importance of reinforcement in driving BPS. In the findings that are discussed, many of the arguments rely on observations from a single bird, Snowball, and little is known about the first six years of his life. His subsequent owners report that he “liked music” from the time that they adopted him, and that they explicitly encouraged this behavior by joining in with arm movements [1]. While this may not constitute “formal” training, it went on for years, and it certainly could be defined as reward for this social species. Indeed, it is worth noting that this is not unlike the way human children learn to move to music: gradually, and with social reward over many years. It therefore seems imperative that assessment of BPS be performed in parrots raised under more controlled conditions. Furthermore, an exclusive focus on spontaneously developing BPS may limit a full comparative understanding of this interesting phenomenon. Reports that animals like sea lions can perform BPS when trained imply that these animals possess the neural substrates for perceiving and synchronizing to beats [2]. We would argue that a focus on spontaneity excludes from consideration (and therefore study) species that don’t display BPS not because they can’t, but because they just don’t.

**Fig. 1:**
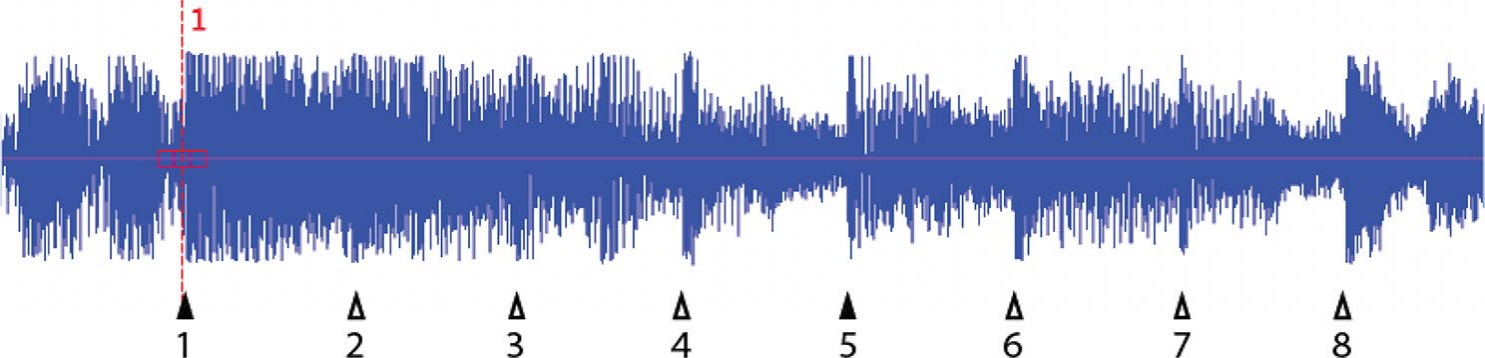
Waveform of 2 bars of “Everybody” by the backstreet boys. First beat of each bar is a filled triangle

One of the critical assumptions underlying BPS is the concept of *complexity* which suggests that a unique feature of parrots and humans is their ability to synchronize to musical beats rather than simply responding to regular amplitude peaks in the music [2]. This ability to perceive a steady beat embedded in a more complex stimulus is typically contrasted with rhythmic abilities of species such as macaques, who can be trained to tap to a metronome but not to music. But is this really a difference in kind, or a difference in degree? Most popular music contains regular peaks in amplitude, at multiple levels of the metrical hierarchy. Depending on the beat, beat perception may be mainly a matter of hearing one or more metronomes in a crowded auditory scene– a kind of “cocktail party problem” for metronomes. The analysis of Snowball’s dancing [1] relied heavily on “Everybody” (by the Backstreet Boys), for example, which has a peak in amplitude on every beat (Fig. 1). For much of the song, the beat is clearly outlined in the peak amplitude, as in beats 3 through 8. In other sections like from the first beat to the third, there is more distracting material interposed, but the beats are still clearly present. In some places, such as in the lead-in to the first beat and between the 3 and 4th beats, another level in the metrical hierarchy is clearly present, dividing each beat into two metrically equal halves.

**Fig. 2:**
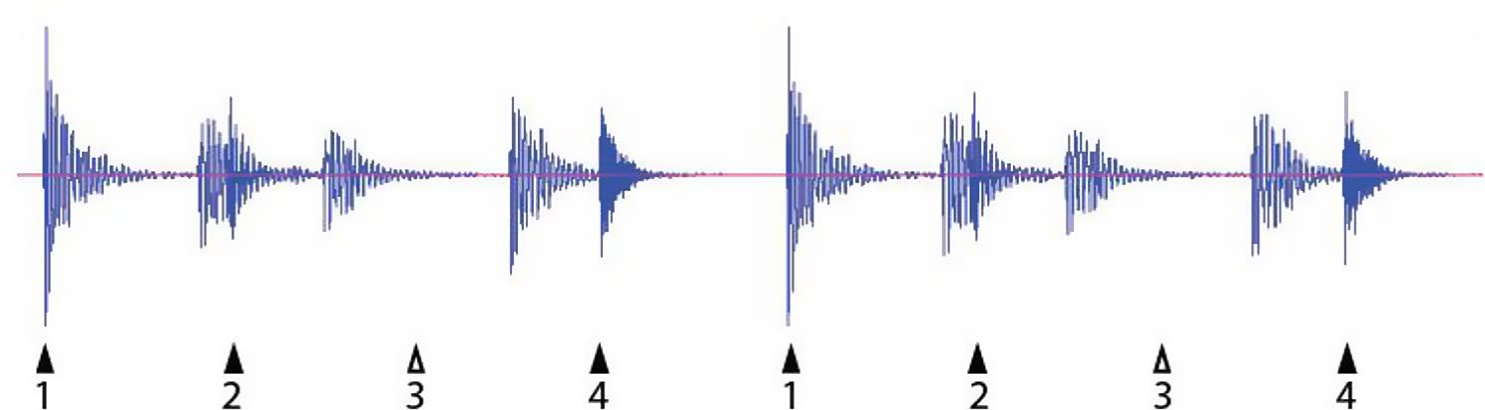
Waveform of two bars of a rhythm where beat 3 *(open triangles)* is implied, but not actually present

None of this is to minimize the importance of BPS in parrots. In fact, it may be worth an even closer look at Snowball’s dance routines, in terms of which sections of the music produce better or worse synchrony. Do Snowball’s “bouts” of accurate synchrony happen in the more “beat explicit” sections of the song, or do they occur equally throughout? Closer analysis of the stimuli might also shed light on the hierarchical nature of BPS. Snowball changed the pattern of his movements several times in each performance, switching his foot stepping from every beat to every other beat, for example. Does this correspond to changes in the stimulus, where different periodicities shifted in relative prominence? Maybe most importantly, would Snowball dance to a more syncopated beat, where (for example) the third beat of every four was implied, but not present? (Fig. 2). Would the implied beat 3 still get a head-bob? This would be a clear distinction from moving to a metronome, where every beat is present. A systematic psychometric approach to these and other features could tell us a lot about the process of beat perception behind BPS.

The observation that BPS appears to be unique among humans and parrots offers an exciting opportunity to initiate comparative studies to probe the neural mechanisms that underlie this behavioral convergence, and this paper suggests an intriguing possibility in the parallel dual pathway neural architecture of vocal learning in both humans and parrots. Because BPS is a complex sensorimotor behavior such studies will need to disambiguate between beat perception (BP) and the ability (or desire) to produce a motor output to these beats that leads to BPS. Are there other ways, besides synchronized movement, to discern whether an animal perceives a given periodicity– a beat– in a musical excerpt? One approach is to simply ask whether an animal can discriminate between rhythms. In an exciting collaboration, Ani Patel and Mimi Kao have started to perform these types of experiments using operant conditioning approaches [3]. They showed that songbirds, which are well-known vocal learners, can discriminate between isochronous and arrhythmic sequences of song vocal elements, a task that non-vocal learners are unable to master. These findings open exciting possibilities for neural circuit-level dissection of this ability. Another possibility would be to utilize the well-established decrease in reaction time for judgments about expected vs. unexpected events. Patel has successfully used this method to investigate harmonically expected vs. unexpected chords in patients with Broca’s aphasia relative to controls [4]. A wide range of animals could be trained on non-musical tasks, which are then presented either on or off the beat of a concurrent audio track. The prediction would be for shorter reaction times for events on the beat, but only in animals that perceive that beat. Another possibility might be to measure breathing patterns. In rodents, repeated stimulation of forebrain inputs to brainstem respiratory control areas can eventually entrain breathing via an NMDA-dependent mechanism [5]. In humans, music is known to entrain breathing, and finger tapping while listening to music enhances the strength of such entrainment [6]. Entrainment of breathing patterns with music might therefore provide a straightforward tool for detecting beat perception in animals. Such entrainment would also be fascinating because rhythmic breathing is known to directly impact brain oscillations [7], which can also have direct effects on emotional states and learning [8].

If such studies suggest that additional animals beyond humans and parrots can perceive musical beats, as is suggested by the songbird studies, then the inability to produce BPS may indeed reflect, as Patel suggests, a lack of functional coupling between the auditory system and parts of the motor system that are capable of producing movements observable as beat synchronization. Alternatively, even if the auditory system is connected to the motor system it might be that it simply is not capable of synchronizing to the range of beat frequencies that parrots and humans can perceive. An interesting example comes from experiments in songbirds where repeated presentations of the bird’s own song drives neural responses in motor areas (and associated muscles) with the stimulus eventually entraining neural responses such that neural activity starts showing anticipatory activation at key transitions in the song [9]. Because neural entrainment only occurs for presentation of the bird’s own song, the inability of zebra finches (who typically only learn a single song) to entrain to a broader range of acoustic signals might be caused by limited sensorimotor coupling to only a single song. In the parrot, which can learn to imitate a wide range of vocalizations throughout its adult life, it is conceivable that similar sensorimotor coupling occurs for all acoustic signals (including song) that it learns.

One of the key features that makes it possible to observe BPS in humans and parrots is behavioral readout. In humans, such readout manifests itself in the form of finger taps, head movements or various forms of dance. In parrots, it manifests as body movement and head bobs [10]. Why parrots should perform such movements is unclear but it might be tied to their natural mating behaviors given that they produce a form of head-bobbing as a prelude to allofeeding [10] (a kind of romantic regurgitation). In both humans and parrots, there might therefore be some natural proclivity to produce these types of movements in synchrony with sound. Species that do not exhibit BPS spontaneously but can be taught to tap to beats or metronomes with reinforcement learning [11, 12] might perceive musical beats but are unable to couple perception into an observable motor output because both system are functionally uncoupled. Perhaps certain forms of reinforcement learning can couple, or unmask latent connections, between auditory circuits for beat perception and motor systems required for observation of beat synchronization.

A final point that should be mentioned in terms of the paucity of observed manifestation of BPS in animals is the relationship between musical beat and reward. Humans (and perhaps, parrots) perform BPS because it is rewarding. In humans, listening to music increases dopamine in dorsal and ventral striatum and this increase in dopamine is proportional with the degree of pleasure derived from the music [13, 14]. Although these studies do not distinguish between music with and without a beat (most music, after all, has a beat), the role of the striatum in prediction and reward suggests that these circuits must be part of any explanation for why we dance, how we dance, and when we dance. Parrots might also experience pleasure in responding to the musical beat, and this might be tied to their behavioral ecology, to the accuracy of their own sensorimotor predictions, or to social reward in the form of approval from their handlers. This raises an important question: does Snowball dance by himself, even when no one is watching?

The observation that parrots, like humans, can perform BPS opens fascinating opportunities for investigating convergent neural mechanisms required for the instantiation of this behavior. It also creates the chance for carefully designed parametrically based comparative, and possibly evolutionary, studies investigating the nature of complex beat perception and how such perception is and can become coupled to synchronization.

## Electronic supplementary material

Below is the link to the electronic supplementary material.

Supplementary Material 1

Supplementary Material 2

## Data Availability

Not applicable.
